# Supplementary material for: MetaRibo-Seq measures translation in microbiomes
Source: Nat Commun. 2020 Jun 29;11:3268. doi: 10.1038/s41467-020-17081-z (PMC7324362; doi:10.1038/s41467-020-17081-z)
Supplement: Supplementary file 10 — Supplementary Data 7 [file 41467_2020_17081_MOESM10_ESM.zip › File2/Confidence_High_Taxonomy/229143_out.krona.html]

Javascript must be enabled to view this page.

members
magnitude
magnitudeUnassigned
count
unassigned
taxon
rank

229143\_out

14

2
14
superkingdom

1239
14
phylum

186801
14
class

order
14
186802

541000
12
family

1
216851
genus


SRS024492\_contig\_number\_580
1
853
species

11

SRS045244\_contig\_number\_8282SRS062654\_contig\_number\_5443SRS142599\_contig\_number\_4696SRS143342\_contig\_number\_207SRS143466\_contig\_number\_contig-100\_856.164586SRS144506\_contig\_number\_31712SRS146888\_contig\_number\_320SRS147139\_contig\_number\_37960SRS148091\_contig\_number\_7057SRS148253\_contig\_number\_1488SRS148511\_contig\_number\_18693
1952288
species

216572
2
family

2
459786
genus

1945593

SRS043768\_contig\_number\_27339SRS063518\_contig\_number\_5647
2
species
